# Advanced glycation end products regulate the receptor of AGEs epigenetically

**DOI:** 10.3389/fcell.2023.1062229

**Published:** 2023-02-14

**Authors:** Xiaoqing Wu, Xuanren Shi, Xiaoyong Chen, Zhanhai Yin

**Affiliations:** ^1^ Department of Orthopaedics, The First Affiliated Hospital of Xi’an Jiaotong University, Xi’an, China; ^2^ Department of Orthopaedics and Traumatology, Shenzhen University General Hospital, Shenzhen, China; ^3^ Department of Hematology and Oncology, Shenzhen University General Hospital, Shenzhen, China

**Keywords:** AGEs, RAGE, DNA methylation, epigenetic regulation, TET1

## Abstract

Advanced glycation end-products (AGEs) can boost their receptor of AGE (RAGE) expression through the downstream signaling pathway to facilitate AGE–RAGE interaction. In this regulation process, the primary signaling pathways are NF-κB and STAT3. However, the inhibition of these transcription factors cannot completely block the upregulation of RAGE, which indicates AGEs may also impact RAGE expression *via* other pathways. In this study, we revealed that AGEs can exhibit epigenetic impacts on RAGE expression. Here, we used carboxymethyl-lysine (CML) and carboxyethyl-lysine (CEL) to treat liver cells and discovered that AGEs can promote the demethylation of the RAGE promoter region. To verify this epigenetic modification, we employed dCAS9-DNMT3a with sgRNA to specifically modify the RAGE promoter region against the effect of carboxymethyl-lysine and carboxyethyl-lysine. The elevated RAGE expressions were partially repressed after AGE-induced hypomethylation statuses were reversed. Additionally, TET1 were also upregulated in AGE-treated cells, indicating AGEs may epigenetically modulate RAGE through the elevating TET1 level.

## 1 Introduction

Advanced glycation end-products (AGEs) are one kind of heterogeneous compounds that are generated from the Maillard reaction. The Maillard reaction is a series of non-enzymatic glycation that occurs between reducing sugars and free amino groups, lipids, or proteins. AGEs are generated either from dietary food processing or metabolized biological activities in normal physiological processes. Long-term intake of AGEs leads to the accumulation of AGEs in our body, which can trigger the outbreak of multiple chronic abnormalities and toxic pathogenesis (Kang and Yang, 2020; Perrone et al., 2020), e.g., diabetes and various diabetic complications. Some AGEs have been chemically well characterized in the body, such as carboxyethyl-lysine (CEL), and carboxymethyl-lysine (CML).

AGE mediated the damage *via* the interaction with its cell surface receptor—RAGE. The binding between AGEs and RAGE can promote the activation of inflammatory response through the NF-κB signaling pathway and cellular apoptosis and meanwhile, also elevate the VEGF level to increase vascular endothelial permeability (Ma et al., 2007; Kang and Yang, 2020). Most importantly, AGEs can also facilitate RAGE upregulation *via* the downstream cellular signal pathways, including NF-κB and STAT3 signaling pathways (Zhou et al., 2020). The AGE–RAGE axis not only brings about the upregulated inflammatory genes expression, but also promotes the positive feedback loops, in which the sustained activation of transcription factors facilitates RAGE expression (Dariya and Nagaraju, 2020). NF-κB is a critical transcription factor for RAGE upregulation in cells (Ott et al., 2014). NF-κB activation is considered an important proinflammatory and proapoptotic signaling to induce AGE-mediated inflammation and apoptosis (Kang and Yang, 2020). RAGE overexpression is also closely associated with the activation of the STAT3 pathway (Abo El-Nasr et al., 2020; Chiappalupi et al., 2020). The interaction of AGEs and RAGE triggers the activation of the STAT3/Pim1/NFAT axis, which can maintain the RAGE high transcription activity by functioning as a positive feedback loop to augment RAGE expression (Meloche et al., 2011).

Epigenetic modification is also a significant regulation for gene expression, especially gene promoter methylation (Dawson and Kouzarides, 2012; Henikoff and Greally, 2016). AGE-mediated epigenetic impacts have been demonstrated to be associated with diabetes and other chronic diseases (Kang and Yang, 2020; Perrone et al., 2020), which are conducted by their interaction with RAGE (Khalid et al., 2022). AGEs can also lead to the downregulation of sirtuin 1 (SIRT1), which provokes high acetylation of the factors, such as STAT3, NF-κB (p65), and FOXO4 (Perrone et al., 2020). Diabetes augments the expression of matrix metalloproteinase (MMP)-9 in the skin and its keratinocytes, and a high level of MMP-9 leads to impairments to skin wound healing, in which AGE-induced demethylation of MMP-9 promoter plays a key role (Perrone et al., 2020). On the other hand, the methylation status in the RAGE promoter region exhibits key effects on multiple diseases (Wang et al., 2019). Previous studies also revealed that RAGE promoter methylation status can function as an indicator for the diabetic retinal inflammation and RAGE gene promoters in DR patients, showing a lower methylation rate than that of healthy adults (Kan et al., 2018). However, few studies have addressed the correlation between AGEs and the status of RAGE promoter methylation. Here, we reveal that AGEs, such as carboxymethyl-lysine and carboxyethyl-lysine, can impact the methylation status of the RAGE promoter and consequently modulate RAGE expression in cells, in which TET1 may play an important role. These results further developed our comprehension of the interaction between AGEs and RAGE.

## 2 Materials and methods

### 2.1 Cell culture and reagents

Human liver cell lines, LO2, were immortal non-tumor cell lines isolated from normal liver tissues. The LO2 cells were cultured in DMEM (Gibco), with the supplementation of 10% FBS and 1% antibiotics (streptomycin-penicillin) at 37°C, in the cell-culture incubator with 5% CO_2_. Cell passaging was performed by trypsin-EDTA solution (0.25%) to digest the cells from the culture dish.

CML and CEL were purchased from Toronto Research Chemicals (Canada). NF-κB and STAT3 inhibitors, niclosamide, and QNZ were purchased from Selleck Chemicals and prepared in DMSO for subsequent dilution to the specific dose in the study.

### 2.2 Cell viability assay

The cell viability was measured by the CCK-8 Reagent (Thermo Scientific) according to the manufacturer’s instructions. Cells were treated for 24 h, washed with PBS, and subsequently added to CCK-8 solution. The absorbance was measured at 450 nm. All experiments were repeated at least three times.

### 2.3 qPCR

Total RNA was extracted using RNAzol (Molecular Research Center, OH, United States) according to the instructions. qPCR was performed with iTaq universal SYBR green supermix (Bio-Rad, Hercules, United States) in a StepOnePlus Real-Time PCR System (Thermo Fisher Scientific). Briefly, the incubation process includes two main steps. Step one is 94°C for 3 min, and step two is 94°C for 10 s and 60°C for 30 s with 40 cycles. The expression of RAGE was calculated by using the 2^−ΔΔCT^ formula. The primers for qPCR were listed in Table 1, and all samples were performed in triplicate.

**TABLE 1:** Details of the primers used in RT-qPCR and sgRNA.

Gene name (RT-qPCR)	Sense (5'-3')		Anti-sense (5'-3')
Tet1	CAG​GAC​CAA​GTG​TTG​CTG​CTG​T		GAC​ACC​CAT​GAG​AGC​TTT​TCC​C
RAGE	CAC​CTT​CTC​CTG​TAG​CTT​CAG​C		AGG​AGC​TAC​TGC​TCC​ACC​TTC​T
GAPDH	AGGTCGGTG TGAACGGATTTG		GGGGTCGTTGATGGC AACA
BSP primers	ATT​TTT​GGA​TAG​AGG​ATA​TGG​G		ATT​CTA​TTA​ATT​TAA​AAT​AAA​CT
		Sequence	
sgRNA-targeting RAGE promoter		TCT​TTC​ACG​AAG​TTC​CAA​AC	
Scrambled sgRNA		CCC​CCG​GGG​GAA​AAA​TTT​TT	

### 2.4 Luciferase reporter assay

NF-κB and STAT3 luciferase reporter plasmids (YESEN, Shanghai, China) were used to test the activity of NF-κB and STAT3. Transient transfection of liver cells was performed using lipofectamine 3000 (Invitrogen, Carlsbad, CA) according to the manufacturer’s instructions. The cells were transfected with the luciferase plasmid. pRL-TK renilla luciferase was used as the internal control. The transfected cells were harvested and lysed according to the protocol of dual-luciferase reporter assay kit (Promega, Madison, WI) to analyze the luciferase activity *via* a luminometer fluorescence reader.

### 2.5 Immunofluorescence assay

The attached cells were fixed by 4% formaldehyde in PBS. After the fix, cells were blocked by 1% BSA. Then, the cells were incubated in the solution of the anti-RAGE primary antibody conjugated to FITC (2 μg/ml) (Santa Cruz Biotechnology), followed by counterstaining with Hochest solution (Invitrogen). Finally, these cells were analyzed under fluorescent microscopy. For flow cytometry analysis, the cells were suspended by trypin and stained as given in the aforementioned protocol.

### 2.6 DNA demethylation blocking, bisulfite conversion, and sequencing

Lentivirus consisting of the sg-RNA-targeting RAGE promoter and dCAS9-DNMT3A (Addgene #84476, #52963) were transducted into cells to specifically prevent DNA demethylation (Kang et al., 2021). Bisulfite modification of DNA was conducted by using EpiTect bisulfite kit (QIAGEN), following the manufacturer’s instructions. Briefly, the genomic DNA were extracted from cells and then incubated with bisulfite mix for 85 min at 60°C. Bisulfated DNA was purified and used as the template for the following PCR and sequencing. The CpG island in the promoter region (Upstream of exon 1 by 1481–1584bp) of modified DNA was amplified by PCR and then purified to sub-clone into the TA cloning vector plasmid. Seven clones in each indicated group were chosen for sequencing randomly. The sequencing results were analyzed in the bisulfite result analysis website: http://quma.cdb.riken.jp/. The information of sgRNA and BSP primers were listed in Table 1.

### 2.7 Statistical analysis

All experiments were conducted in at least three independent trials. Student’s t-test was used for the analysis. *p*-value less than 0.05, 0.01 and 0.001 were presented as “*,” “**,” and “***,” respectively. *p*-values less than 0.05 were regarded as being significant statistically.

## 3 Results

### 3.1 CML and CEL augment the expression of RAGE in LO2 cells

**FIGURE 1:**
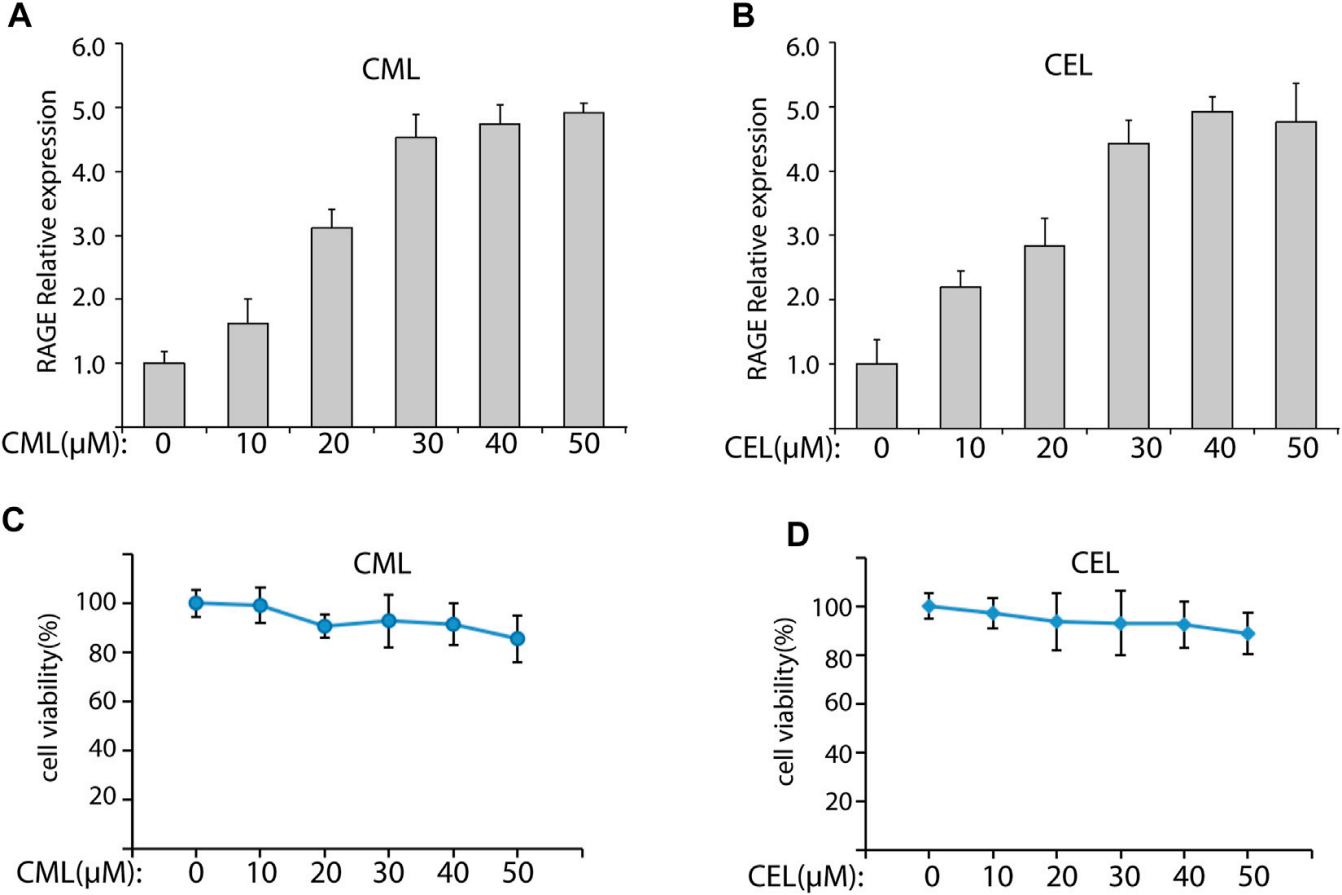
CML and CEL can upregulate RAGE expression in LO2 cells. **(A,B)** RAGE expression in cells with different treatment doses of CML and CEL for 24 h. **(C,D)** Effects of different doses of CML and CEL on cell viability. All dates are shown as the mean ± SD.

**FIGURE 2:**
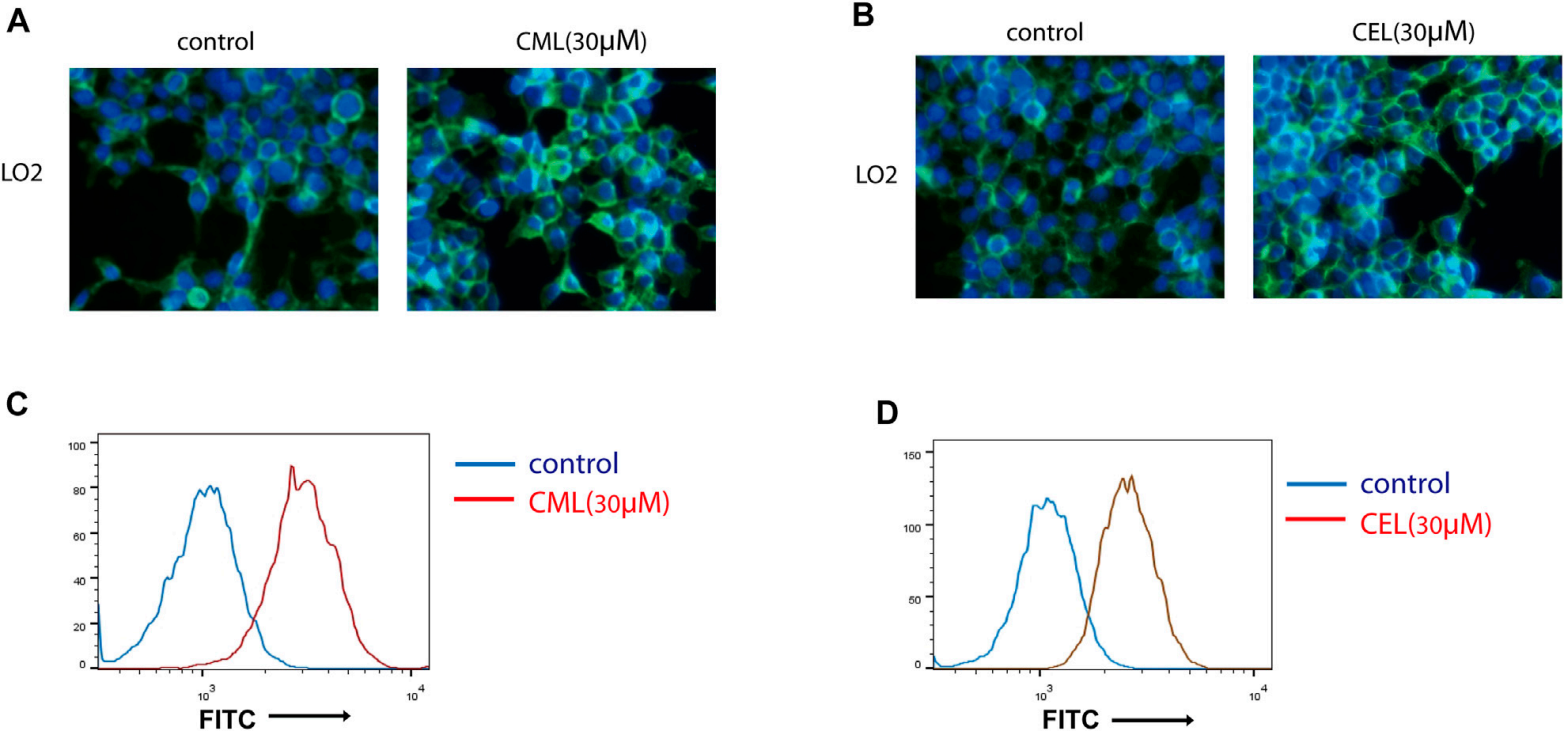
Expression of RAGE were detected by immunofluorescence assay. Control and CML/CEL cells were stained with an antibody of anti-RAGE-FITC and Hoechst 33342, and examined by fluorescence microscopy **(A,B)** and flow cytometry **(C,D)**.

To evaluate the modulation of CML and CEL on RAGE expression, we incubated LO2 cells with different concentrations of CML and CEL to detect the expression of RAGE, respectively. It was found that 10–50 μM CML or CEL dose-dependently increased cellular RAGE expression, which further increased up to 30 μM and did not bring about any stronger expression of RAGE (Figures 1A, B). The RAGE expression was concentration dependent associated with AGEs—CML and CEL. To preclude the interference from the potential effects of CML and CEL on the viability of LO2 cells, we conducted CCK-8 assay with a range of above mentioned concentrations. CML and CEL showed weak influence on the LO2 cell lines (Figures 1A, B). Hence, 30 μM (non-cytotoxic concentration) of CML or CEL was adopted in subsequent experiments. The positive modulation of RAGE by CML and CEL in LO2 cells was also confirmed by fluorescence microscopy and flow cytometry analysis. The RAGE on the cell membrane was stained by antibody conjugated with FITC. Compared with the control, CML/CEL-treated cells exhibit stronger florescence intensity (Figures 2A, B), directly manifesting the upregulation of RAGE, which also confirmed by the flow cytometer (Figures 2C, D).

### 3.2 RAGE upregulation by CML and CEL is not mediated solely through NF-κB and STAT3

Previous studies have revealed that the AGE–RAGE axis in the intracellular and extracellular signal transduction eventually give rise to the activation of NF-κB and STAT3. The activation of NF-κB and STAT3 triggers the successive transcription of RAGE and further boosts various RAGE-reliant signaling pathways. These two factors play a critical role in the vicious cycle. To clarify whether the activation of NF-κB and STAT3 is the sole modulation for CML/CEL-mediated RAGE upregulation, niclosamide and QNZ, widely used STAT3 and NF-κB specific inhibitors, were used to see if it could completely block the effect produced by these AGEs.

**FIGURE 3:**
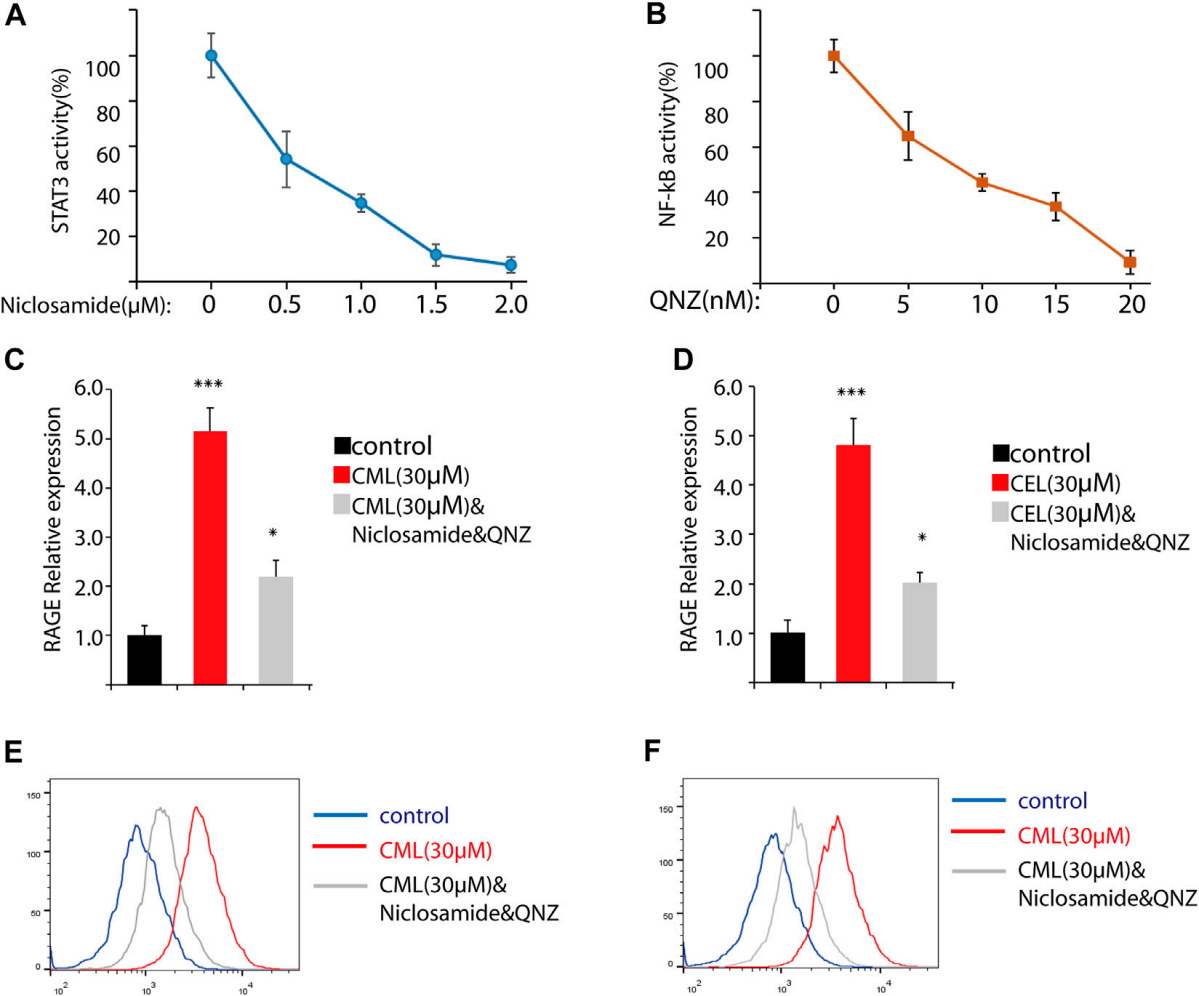
Inhibition of NF-κB and STAT3 cannot completely block RAGE upregulation in cells. **(A,B)** Inhibition of NF-κB and STAT3 by their inhibitors. **(C,D)** RAGE expression of the indicated treatment in cells. **(D,E)** Flow cytometry analysis of RAGE expression. All dates are shown as the mean ± SD. *p < 0.05, ***p < 0.001.

STAT3 and NF-κB luciferase reporter plasmids were used to detect the inhibitory effect of the drug. These two transcription factors’ activity was repressed dose-dependently (Figures 3A, B). Here, we chose 2 μM and 20 nM for STAT3 and NF-κB inhibition in the following study.

qPCR analysis found STAT3 and NF-κB inhibitors significantly repressed RAGE levels in CML and CEL treated cells, while these inhibitors did not completely block the CML/CEL-induced upregulation of RAGE (Figure 3C, D), and the flow cytometry of the cell stain with the anti-RAGE antibody conjugated with FITC also showed the similar results (Figure 3E, F), suggesting the existence of an NF-κB/STAT3-independent mechanism underlying CML/CEL’s effect.

### 3.3 AGEs can regulate RAGE expression epigenetically

**FIGURE 4:**
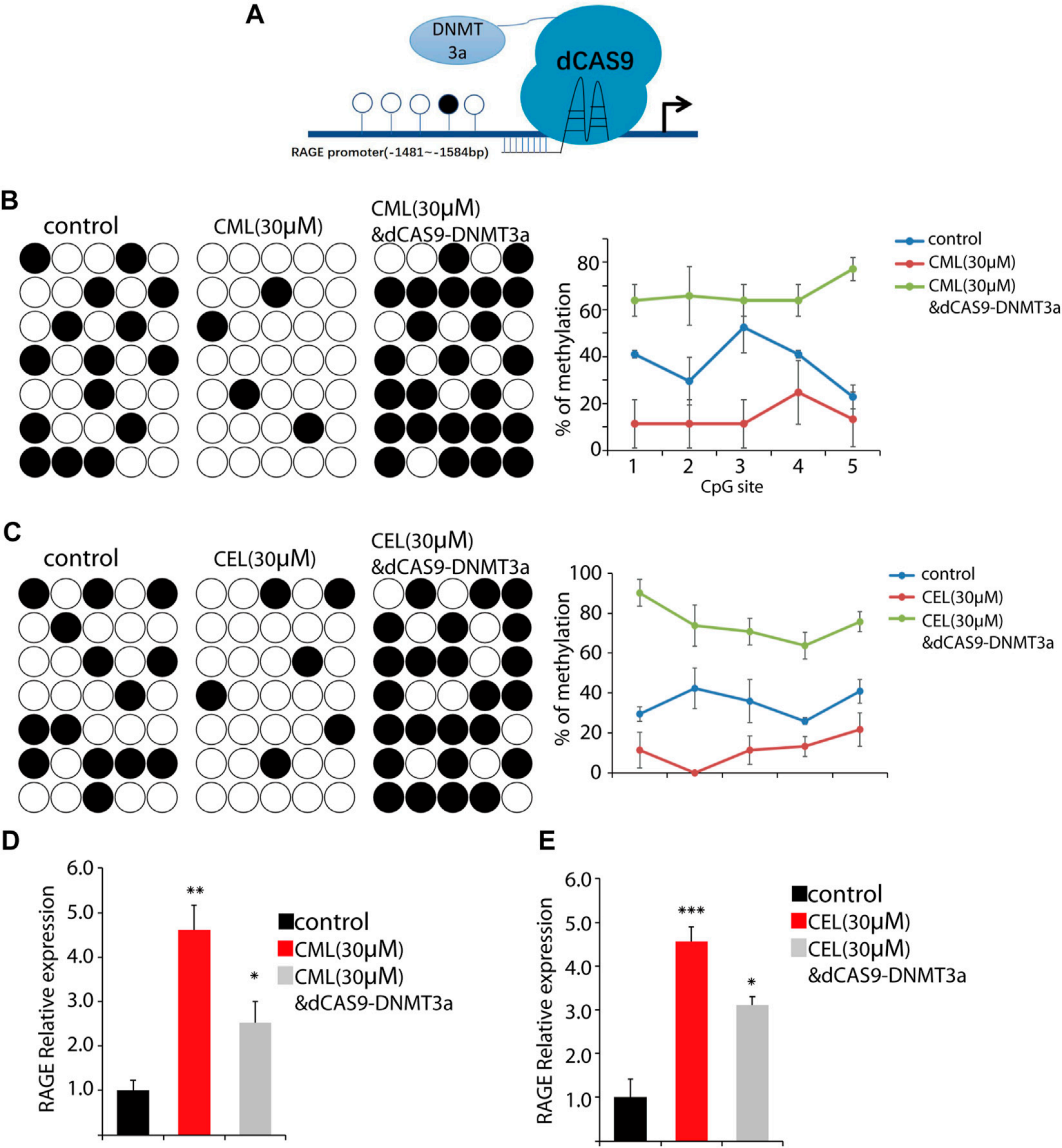
Change of the RAGE promoter methylation status. **(A)** Representative images of dCas9-DNMT3a-mediated specific modification. **(B,C)** Changes in the methylation status of the RAGE promoter region. CML and CEL enhanced the demethylation level of the RAGE promoter, and the dCas9-DNMT3a-targeting RAGE promoter recovered the hypermethylation status. **(D,E)** Relative expression of RAGE in the indicated groups. All dates are shown as the mean ± SD. *p < 0.05, **p < 0.01, ***p < 0.001.

The RAGE gene promoter methylation status is closely associated with AGE-induced chronic disease, such as diabetic complications (Kan et al., 2018), and promoter hypo-/hypermethylation acts as an important epigenetic modulation for gene expression. Having established AGE-induced concentration-dependent upregulation of RAGE expression in cells, we sought to determine their impact on the methylation status in the RAGE promoter region. Here, we used CML and CEL for the following methylation study. To conduct the epigenetic modification at a specific site, we use the dCAS9-DNMT3a to mediate methylation in the RAGE promoter region (Figure 4A), and BSP were preformed to detect the change of the methylation status. The BSP results revealed that CML and CEL prompt the hypomethylation status in the RAGE promoter region, and the average methylation rate of individual CpGs decreased by 23% and 25%, respectively (Figures 4B, C).

To verify this phenomenon, dCAS9-DNMT3a- and sgRNA-targeting RAGE promoter regions were transfected into the cells to counteract the effect of CML/CEL. As expected, CML/CEL-mediated demethylation was abrogated by the dCAS9-DNMT3a-mediated methylation, and the increased fold change of the average DNA methylation rate of individual CpGs between control and dCas9-DNMT3a-treated cells is about 30% (Figures 4B, C). Correspondingly, the dCAS9-DNMT3a-targeting RAGE promoter region partially blocked the CML/CEL-mediated augment of RAGE expression (Figures 4D, E), while the scrambled sgRNA with dCAS9-DNMT3a exhibited no effect on RAGE expression (Supplementary Figure S1).

### 3.4 AGEs upregulate TET1 expression

**FIGURE 5:**
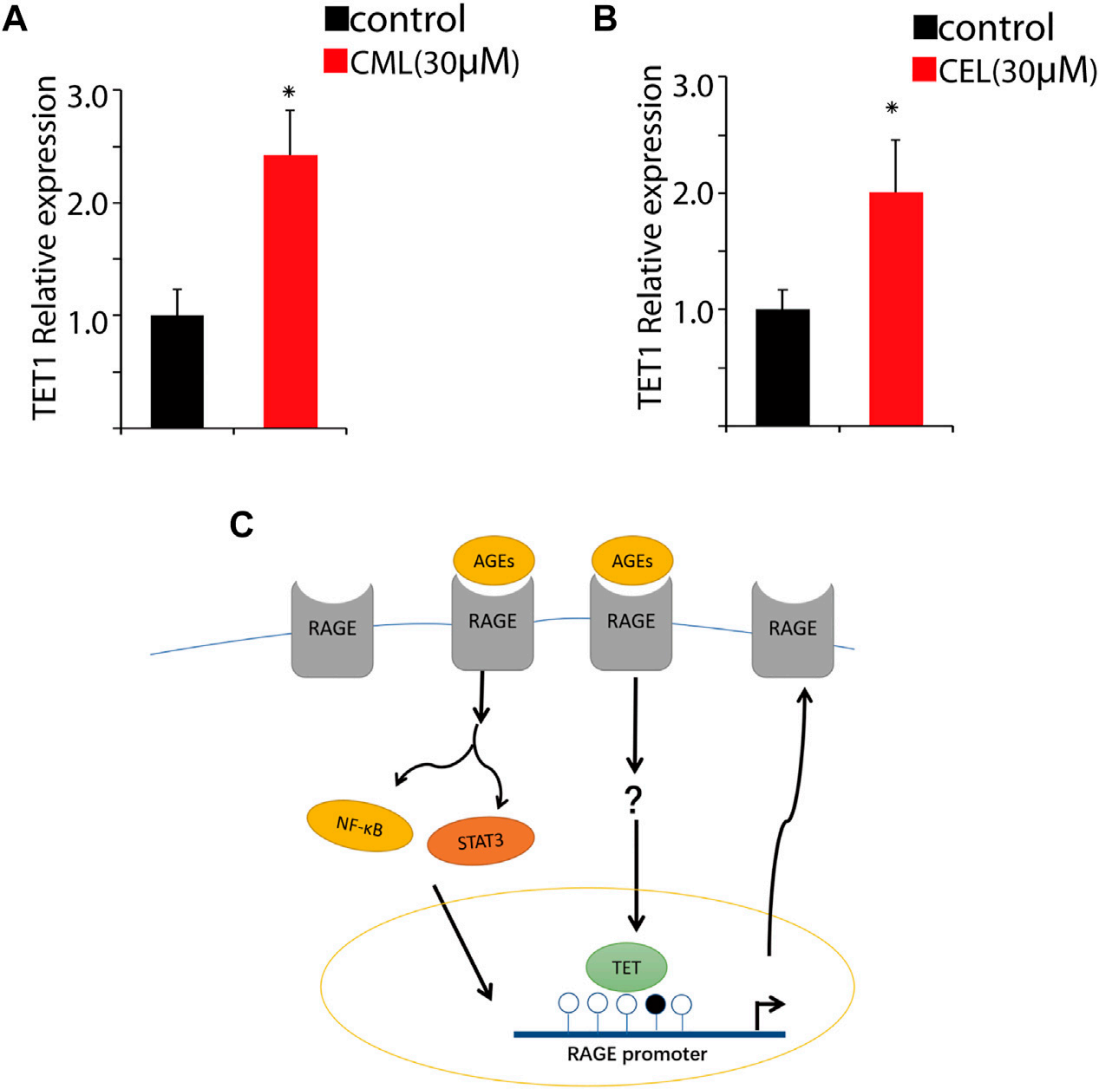
Change of the RAGE promoter methylation status. Tet1 expression affected by CML **(A)** and CEL **(B)**. **(C)** Representative images of the regulatory process. All dates are shown as the mean ± SD. *p < 0.05.

DNA demethylation is mainly conducted by TET1 that is a maintenance DNA demethylase to prevent methylation spreading in cells. Here, we performed qPCR to analyze the impact of CML and CEL on TET1 expression. Our results revealed that the treatment of CML and CEL upregulates TET1 expression (Figures 5A, B), which indicates AGE and RAGE interactions may facilitate the elevation of TET1 level to mediate epigenetic modulation of RAGE. As Figure 5C shows, the interaction between AGEs and RAGE not only activates its downstream NF-κB/STAT3 signaling pathway to augment RAGE expression as a feedback, but also facilitate the demethylation of the RAGE promoter region to boost its transcription. However, how AGEs upregulate TET1 expression still remains unknown.

## 4 Discussion

It is well-known that AGEs can promote RAGE expression through the downstream pathway—NF-κB and STAT3 signaling pathways, to augment the interaction between AGEs and RAGE. In this study, we further discovered AGEs can also epigenetically modulate RAGE expression, which deepens our understanding of this feedback loop. AGEs and their receptor RAGE play critical roles in the progression of chronic disease, such as diabetes and cancer (Sanajou et al., 2018). Notably, the AGE–RAGE axis can activate its downstream signaling to facilitate RAGE expression, which forms a vicious cycle to escalate its impact. In this cycle, the key factor is NF-κB and STAT3 (Fuentes et al., 2007; Oh et al., 2019). However, their inhibitors cannot completely block RAGE upregulation, indicating that other pathways exist in this regulatory phenomenon.

Epigenetic modification is an important modulation of gene expression, which mediates the dynamic processes of transcriptional activation or suppression without the alteration of the DNA sequence (Afanas’ev, 2014; Hogg et al., 2020). The AGE-mediated epigenetic effects have been revealed to involve the occurrence of diabetes and other chronic diseases. Recent studies conducted in human podocytes showed that AGEs enhanced the acetylation of key transcription factors *via* the downregulation of SIRT1, leading to podocyte apoptosis and consequently kidney diseases (Perrone et al., 2020). AGEs induced the demethylation of the MMP-9 promoter through the downregulation of GADD45a, which is involved in diabetic foot ulcers (Zhong and Kowluru, 2013; Zhou et al., 2018).

DNA methylation has been recently demonstrated to be involved in glycemic memory of diabetic complication (Tewari et al., 2012; Dunn et al., 2014), the epigenetic changes were sustained even if blood glucose return to normal levels (Park et al., 2014; Perrone et al., 2020). DNA methylation also plays an important role in the progression of diabetic foot ulcers. Global hypomethylation is evident in diabetic foot ulcer fibroblasts. Functional enrichment analysis in the previous study emphasized differential methylation of gene clusters that are associated with the myofibril function, angiogenesis, and extracellular matrix for the wound healing process. (Park et al., 2014). Notably, the methylation status of the RAGE promoter can also perform as indicator for diabetic complication (Rajasekar et al., 2015). Kan and colleagues have proved the association between RAGE promoter methylation and diabetic retinal inflammation, and the hypomethylation of RAGE promoter can raise IL-1β, IL-6, and TNF-α levels in serum of patients (Kan et al., 2018), and the augmentation of RAGE promoter methylation may contribute to reducing the inflammation of DR patients. Moreover, the promoters of RAGE glomeruli from diabetic db/db mice showed enhanced RNA polymerase II recruitment, increased levels of activated marks and decreased levels of repressive marks (Reddy et al., 2014). Thus, the regulation of methylation in the RAGE promoter region provides an optional way to control these chronic diseases.

It is well-known that AGEs can promote RAGE expression to form the feedback cycle and amplify the influence of their interaction, while the epigenetic relationship between AGEs and RAGE remains elusive. AGEs have an environmental influence, and their epigenetic effects on the downstream genes mainly rely on their interaction with RAGE. Based on the AGE-mediated epigenetic action and RAGE promoter methylation changes, we accordingly proposed the hypothesis that whether AGEs can exhibit epigenetic effects on RAGE transcription to facilitate RAGE expression in this vicious cycle. Here, we identified the epigenetic relationship between AGEs and RAGE promoter methylation and demonstrated this association *via* the dCAS9-DNMT3a system. Meanwhile, we also found out that AGEs can elevate the TET1 levels in the cells, which may contribute to hypomethylation of the RAGE promoter. However, the exact mechanism for this process still remains unknown, and it is also worth exploring the roles of this epigenetic regulation in chronic disease, such as diabetes and cancers, for better therapy.

The damages mediated by the AGE and RAGE axis occur in multiple diseases (Kang and Yang, 2020; Corica et al., 2019; Cai et al., 2004). The binding of AGEs and RAGE induces the activation of multiple downstream signaling pathways, which are associated with numerous cellular processes of inflammation, vasculopathy, apoptosis, nephropathy, and angiogenesis (Ott et al., 2014). Although NF-κB pathway and STAT3 pathway play primary roles in AGE-induced RAGE upregulation, there may still be other unclear downstream signaling pathways in this modulation process. Moreover, with regard to promoter methylation, there may be other factors such as environmental influences to modulate the methylation status. For example, disturbed blood flow has been identified to alter DNA methylation patterns in murine arterial endothelial cells in a DNMT-dependent manner (Dunn et al., 2014).

In conclusion, the present study demonstrates that AGEs can modulate RAGE expression not only through its downstream signaling pathway, but also through the epigenetic modifications. The interaction of AGEs and RAGE induces the demethylation of the RAGE promoter possibly *via* upregulating the TET1 level. However, the exact mechanism underlying these epigenetic regulatory processes still needs to be explored.

## Data Availability

The original contributions presented in the study are included in the article/Supplementary Material; further inquiries can be directed to the corresponding author.
