# Supplementary material for: Advanced glycation end products regulate the receptor of AGEs epigenetically
Source: Front Cell Dev Biol. 2023 Feb 14;11:1062229. doi: 10.3389/fcell.2023.1062229 (PMC9971228; doi:10.3389/fcell.2023.1062229)
Supplement: Supplementary file 1 [file Table1.DOCX]

Table 1. Details of the primers used in RT-qPCR and sgRNA.

| Gene name  (RT-qPCR) | Sense (5'-3') | | Antisense (5'-3') |
| --- | --- | --- | --- |
| *Tet1* | CAGGACCAAGTGTTGCTGCTGT | | GACACCCATGAGAGCTTTTCCC |
| *RAGE* | CACCTTCTCCTGTAGCTTCAGC | | AGGAGCTACTGCTCCACCTTCT |
| *GAPDH* | AGGTCGGTG TGAACGGATTTG | | GGGGTCGTTGATGGC AACA |
| *BSP primers* | ATTTTTGGATAGAGGATATGGG | | ATTCTATTAATTTAAAATAAACT |
|  | | sequence | |
| *sgRNA targeting RAGE promoter* | | TCTTTCACGAAGTTCCAAAC | |
| *scrambled sgRNA* | | CCCCCGGGGGAAAAATTTTT | |
