# Supplementary material for: Advanced glycation end products regulate the receptor of AGEs epigenetically
Source: Front Cell Dev Biol. 2023 Feb 14;11:1062229. doi: 10.3389/fcell.2023.1062229 (PMC9971228; doi:10.3389/fcell.2023.1062229)
Supplement: Supplementary file 2 [file DataSheet1.PDF]

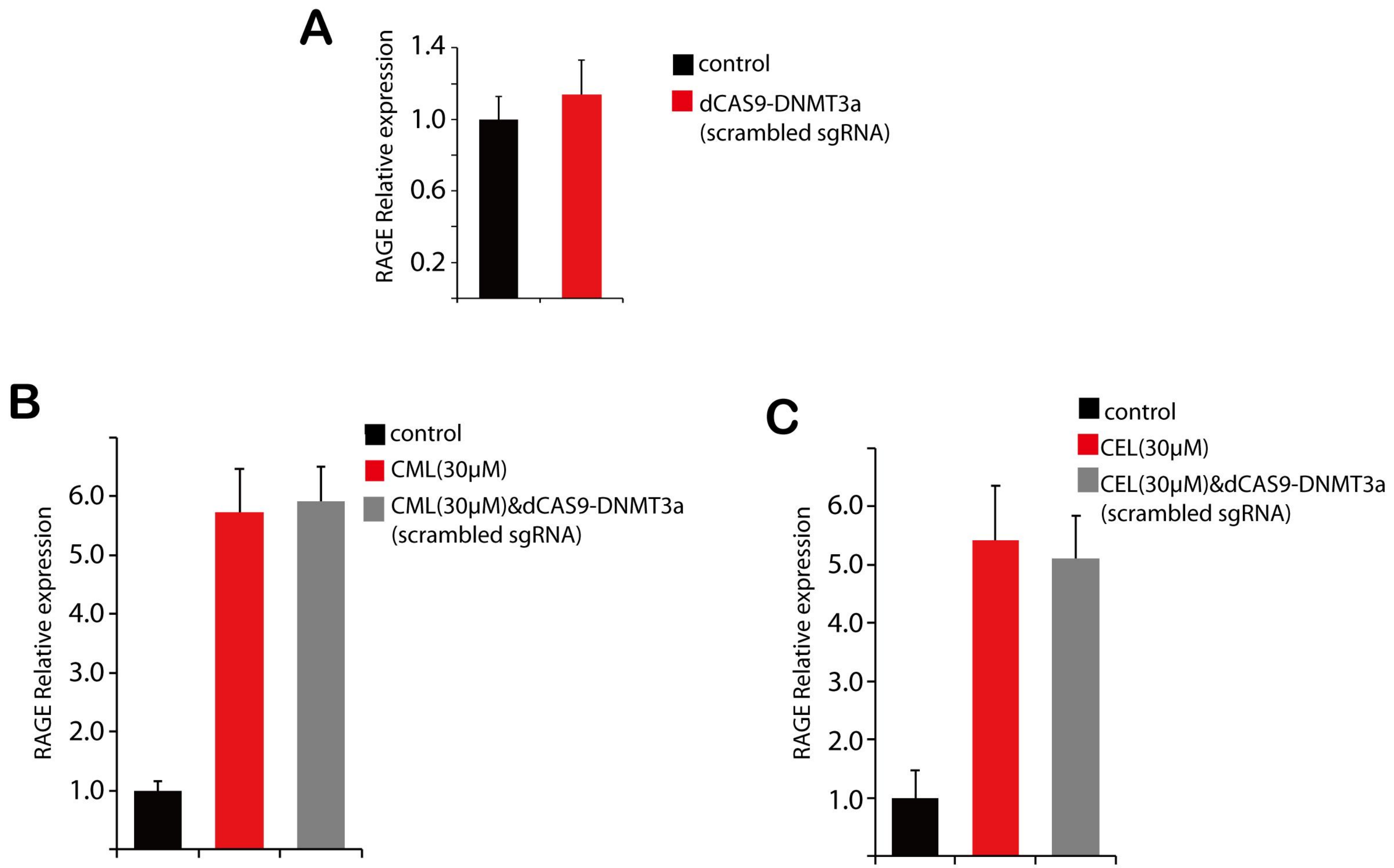

Supplementary Figure 1：(A) dCAS9-DNMT3a with scrambled sgRNA exerts no effects on RAGE expression.
(B and C) dCAS9-DNMT3a with scrambled sgRNA can not block the upregulation of RAGE incued by CML or CEL.
